# FROM CRISIS TO CONTROL THROUGH COVID-19: NURSING HOME LEADERSHIP PERSPECTIVES ON DELIVERING QUALITY CARE

**DOI:** 10.1093/geroni/igad104.2021

**Published:** 2023-12-21

**Authors:** Liza Behrens, Emily Heilbrunn, Laruen Van Scoy, Ashmita Grewal, Nicole Osevala, Janice Whitaker, William Calo, Jennifer Kraschnewski

**Affiliations:** Pennsylvania State University, State College, Pennsylvania, United States; Penn State College of Medicine, Hershey, Pennsylvania, United States; Pennsylvania State University, Hershey, Pennsylvania, United States; Pennsylvania State University, Hershey, Pennsylvania, United States; Pennsylvania State University, Hershey, Pennsylvania, United States; Nese College of Nursing, Penn State University, State College, Pennsylvania, United States; Penn State College of Medicine, Hershey, Pennsylvania, United States; Penn State College of Medicine, Hershey, Pennsylvania, United States

## Abstract

A year after the US Government announced that the COVID-19 pandemic has transitioned to an endemic phase, nursing home (NH) leaders are still challenged to deliver quality care. This study aimed to describe NH leadership perspectives on preparing and maintaining quality care during times of diminishing resources as experienced through the COVID-19 pandemic to gain a perspective on how best to support NHs moving forward. We used convergent mixed methods to explore experiences of NH leaders (N=15) participating in the Project ECHO (Extension for Community Health Outcomes) study. Data were collected through publicly reported survey data and individual interviews conducted at 12-month follow-up. Quantitative data were analyzed using descriptive statistics. Qualitative data were analyzed using verbatim transcripts and inductive thematic coding subsequently organized by a guiding framework. Convergent analysis indicates that participating NHs (N=136) align with national trends in infection rates of residents, staff, access to resources such as personal protective equipment and testing supplies. Leadership from a sub-sample of these homes (N=14; 43% rural; 71% not-for-profit) perceived their NH to be in varied states of operations needed so support quality infection control at the transition to endemic COVID-19 phase. Leadership reported continued challenges in addressing resident & staff vaccinations, securing testing supplies, obtaining financial resources to maintain acceptable levels of personal protective equipment, and isolation practices. Leadership valued providing person-centered care even in times of crisis. These results indicate socio-cultural reasons behind why NHs continue to struggle delivering quality care post-pandemic and offers areas to focus support efforts.

